# Effects of Frost Damage and Nanomaterials Modification on the Microstructure and Fracture Properties of the Interfacial Transition Zone of Cementitious Materials

**DOI:** 10.3390/nano15211670

**Published:** 2025-11-03

**Authors:** Xiangong Zhou, Xiancheng Zhou, Weikang Kong

**Affiliations:** 1 School of Urban Construction, Hangzhou Polytechnic, Hangzhou 311402, China; zhouxiangong@163.com; 2 College of Architectural Engineering, Shanxi Vocational University of Engineering Science and Technology, Jinzhong 030619, China; zhouxiancheng@sxgkd.edu.cn; 3 Intelligent Transportation System Research Center, Southeast University, Nanjing 210096, China

**Keywords:** cementitious composite materials, fracture toughness, nanoscratch technique, nano-silica, interfacial transition zone

## Abstract

Cementitious materials are multiscale and multiphase composites whose frost resistance at the macroscale is closely governed by microstructural characteristics. However, the interfacial transition zone (ITZ) between clinker and hydrates, recognized as the weakest solid phase, plays a decisive role in the initiation and propagation of microcracks under freezing conditions. Understanding the frost damage mechanism of ITZ is therefore essential for improving the durability of concrete in cold regions. The motivation of this study lies in revealing how freezing affects the mechanical integrity and microstructure of ITZ in its early ages, which remains insufficiently understood in existing research. To address this, a nanoscratch technique was employed for its ability to quantify local fracture properties and interfacial adhesion at the submicronscale, providing a direct and high-resolution assessment of ITZ behavior under freeze–thaw action. The ITZ thickness and fracture properties were characterized in unfrozen cement paste and in cement paste frozen at 1 and 7 days of age to elucidate the microscale frost damage mechanism. Moreover, the enhancement effect of nano-silica modification on frozen ITZ was investigated through the combined use of nanoscratch and mercury intrusion porosimetry (MIP). The correlations among clinker particle size, ITZ thickness, and ITZ fracture properties were further established using nanoscratch coupled with scanning electron microscopy (SEM). This study provides a novel micromechanical insight into the frost deterioration of ITZ and demonstrates the innovative application of nanoscratch technology in characterizing freeze-induced damage in cementitious materials, offering theoretical guidance for designing durable concrete for cold environments.

## 1. Introduction

In cold and high-altitude regions, frost damage is a critical factor contributing to the cracking and erosion of concrete structures. Repeated freezing and thawing cycles can lead to the propagation of cracks in concrete, resulting in the deterioration and eventual failure of the concrete. The deterioration of concrete under freeze–thaw cycles is primarily caused by the expansion of pore water upon freezing. If the concrete lacks sufficient strength to resist the tensile stress caused by expansion, irreversible tensile deformation and randomly distributed microcracks will develop at the macroscale [1,2]. Therefore, the frost damage to concrete can be characterized as a process of complex crack propagation [3], and fracture mechanics provides a valuable framework for understanding the development of cracks in concrete under freezing conditions.

Concrete is a multiscale and multiphase composite material, with each phase contributing differently to the overall fracture properties of concrete. At the macroscale, Kosior-Kazberuk [4] studied the deterioration of concrete fracture energy under frost damage by modeling the post-peak load-deflection response of concrete subjected to various freeze–thaw cycles, thereby formulating a computational approach for fracture energy quantification. Ma et al. [5], Li et al. [6], and Cai et al. [7] compared the fracture properties of ordinal concrete and slag concrete subjected to freeze–thaw cycles. These studies consistently reported a pronounced reduction in fracture toughness with increasing freeze–thaw cycles, and slag concrete exhibits superior fracture properties compared to ordinary concrete under the same freeze–thaw conditions. Dong et al. [8] studied the evolution process of frost damage and concrete fracture energy subjected to early age freezing, concluding that the development of cracks caused by freeze–thaw cycles was identified as the main cause of reduced fracture performance. Li et al. [9] studied the crack propagation and concrete fracture properties evolution during freeze–thaw cycles. It is shown that 30 freeze–thaw cycles could reduce the fracture performance of concrete by 60% and significantly reduced the critical stress required for crack initiation.

At the mesoscale of concrete, crack development predominantly occurs at the aggregate–mortar interface [10]. The ITZ thickness exhibits significant dependence on both the aggregate size and the water-to-cement (*w*/*c*) ratio of the concrete [11,12,13]. The thickness of ITZ around aggregate is about 10~50 μm, exhibiting lower strength than cement paste and containing numerous microcracks. Generally, a higher *w*/*c* ratio results in increased porosity and a greater number of cracks in the ITZ region, leading to reduced fracture energy. Prokopski and Langier [14] studied concrete with *w*/*c* ratios of 0.33~0.66 and found that the concrete with lower *w*/*c* ratio exhibits higher fracture toughness. Chen et al. [15] found that in low-strength concrete, the ITZ between aggregate and cement paste serves as the primary zone for crack propagation. As aggregate size increases, the crack propagation path lengthens, thereby increasing the fracture energy of concrete to some extent. However, in high-strength concrete, the improved homogeneity results in similar strengths between aggregate and mortar, reducing the probability of crack propagation along the ITZ. Cracks are more likely to propagate through the fine aggregate, contributing to an increase in the fracture energy of concrete [16].

All these studies confirm that the ITZ plays a dominant role in determining the fracture performance of concrete. However, current research has primarily focused on the macro or mesoscale behavior of ITZ, with very limited understanding of its microfracture characteristics under freeze–thaw conditions. In particular, the fracture behavior of the ITZ between unhydrated clinker and hydration products, the intrinsic microstructural weak phase of cementitious materials, remains largely unexplored. This knowledge gap restricts the mechanistic understanding of frost damage from a microstructural and micromechanical perspective. Compared to the ITZ around large aggregates, the size of the ITZ around the clinker could exist at nano or submicroscales. Measuring this interface requires either a series of individual indents across the interface or a method of recording data continuously across the interface. The nanoscratch test is mainly applied to detect the total width of the interface because it provides continuous collection of the data during testing. Thus, information about the fine structures and mechanical properties (e.g., tribological behavior) of materials can be obtained by a nanoscratch test.

Over the past two decades, nanoscratch technology has been used for nano and microscale material characterization. Ulm et al. [17,18,19] proposed equations for calculating fracture toughness based on scratch tests within the framework of linear-elastic fracture mechanics, significantly advancing the methods available for studying fracture properties at the microscale [20,21,22]. Meanwhile, Wei et al. [23] developed related techniques for phase identification and an ITZ thickness calculation, which is proposed by the fracture toughness distribution and has also been widely applied in the study of microfracture mechanics of cementitious materials. Compared to traditional indentation or tensile testing methods, nanoscratch enables continuous, high-resolution measurement of local fracture responses along the ITZ, making it particularly suitable for investigating microscale interfaces affected by freezing.

With the growing interest in the application of nanomaterials in engineering, nanomaterials such as nano-silica have been explored for enhancing the frost resistance of cementitious materials. Gonzalez et al. [24] incorporated nano-silica into various types of concrete and observed the surface morphology of concrete using scanning electron microscopy after freeze–thaw cycles. It is found that the nano-silica could enhance the density and reduce the permeability of concrete, thereby reducing the frost damage. Cheng et al. [25] conducted research on the frost resistance of concrete mixed with nano-silica, demonstrating that nano-silica can increase the content of hydrated calcium silicate inside the concrete and improve its durability. Wang et al. [26] explored the improvement mechanism of nano-silica on the durability of high-performance concrete. It was found that the addition of nano-silica can effectively enhance the impermeability of high-performance concrete, leading to the improved freeze–thaw durability of concrete. Zhao et al. [27] studied the relationship between the frost resistance of nano-silica-strengthened concrete and the air content and found that the frost resistance of the nano-silica-strengthened concrete could be improved when increasing the air content. Nevertheless, most existing studies have not addressed how nano-silica modifies the ITZ fracture behavior at the microscale under freezing conditions. Therefore, the fundamental mechanism by which nano-silica enhances frost resistance from a fracture mechanics perspective remains unresolved.

In this study, to reveal the frost damage mechanism of cementitious materials and the enhancement mechanism of nano-silica on the frost resistance of cementitious materials, the microfracture properties of ITZ between clinker and hydrates in frozen concrete was tested and calculated by nanoscratch technology at the microscale. The thickness of the ITZ was quantified, the relationship between ITZ thickness and fracture properties was analyzed, and the effects of clinker size on ITZ thickness and fracture properties were explored. This study provides the quantitative evaluation of ITZ fracture behavior in frozen cement paste at the microscale, and demonstrates the synergistic application of nanoscratch, MIP, and SEM techniques to elucidate the microstructural mechanism of frost resistance enhancement by nano-silica.

## 2. Experimental

### 2.1. Materials and Sample Preparation

The cement paste sample was prepared by using ordinary Portland cement, and the *w*/*c* ratios was set to 0.3 and 0.5 to represent the concrete materials with low and high *w*/*c*. Table 1 shows the chemical composition of cement used in this study. To study the improvement of nanomaterials on the microstructure and micromechanical properties of frozen cementitious materials, nano-silica (NS) particles were incorporated by substituting 2% of cement weight, and the proportion was adopted according to the previous studies [28,29,30]. The properties of NS particles are also listed in Table 1.

**Figure 1:**
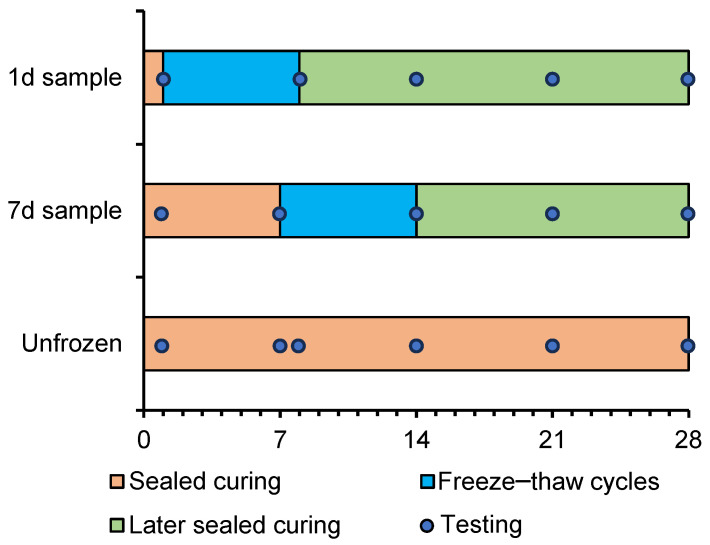
Duration of freeze–thaw cycles, sealed curing, and the age when scratching testings were conducted.

After mixing nano-silica with cement paste, fresh paste was poured into a 25 mm diameter and 100 mm height plastic tube. It was then sealed and cured at 20 °C until the freeze–thaw cycles began. The freezing conditions in this tests were designed to replicate dry and cold environments found in high-altitude regions [31,32]. Each freeze–thaw cycle was completed within six hours, with temperature variations between −20 °C and 20 °C at a constant rate. The curing scheme is shown in Figure 1. The freeze–thaw cycles were initiated at 1 and 7 days of age for the cement paste, respectively, examine early age frost effects. The total freezing duration for each sample was 7 days, thus amounting to 28 cycles in total. After completing the freeze–thaw test, the samples were sealed again and cured at room temperature until reaching an age of 28 days. The purpose of the secondary curing was to assess the recovery of frost damage in the mechanical properties and microstructure.

An unfrozen sample, which was not subject to freeze–thaw cycles and was cured during the entire time under the sealed condition, was also prepared for each mixture and tested for comparison. Table 2 presents the proportioning and naming of the paste sample. “OP” refers to the pure ordinary cement paste sample, “NS” refers to the cement paste sample mixed with nano-silica particles, the subscripts “0.3” and “0.5” represent the *w*/*c* ratio, and “1d” and “7d” represent the samples subject to freeze–thaw cycles at the age of 1 day and 7 days.

After the secondary sealed curing was completed, the 25 mm diameter sample was then cut into 20 mm height cylinders at the middle part of the 100 mm height sample. Due to the bleeding phenomenon, the sample in the middle section exhibited greater uniformity. To meet the requirements for micro testing, grinding and polishing processes were conducted on the samples to obtain a smooth surface with a roughness less than 40 μm. The polishing process was carried out following the methodology described in the literature [33].

### 2.2. Nanoscratch Test

Scratching testing was conducted by a KEYSIGHT G200 nano indenter (Keysight, Colorado Springs, CO, USA) in scratching mode with a Berkovich probe. Following the methodology proposed by Wei et al. [23], a constant vertical loading speed mode was adopted, with a lateral scratching speed of 1 μm/s, a maximum vertical load of 10 mN, and a scratching length of 100 μm. The beginning of each scratching was positioned in the clinker phase with a distance of 50 μm from the ITZ, which ensured that all phases could be scratched within the scratching length of 100 μm. In each nanoscratch test, data were recorded at equal intervals of 1 μm, so that 101 data points could be recorded for each scratch.

From the nanoscratching test, the scratching depth (*d*) and the lateral scratching force (*F_T_*) can be directly tested, which can be used to calculated the fracture toughness (*K_C_*) and the fracture energy (ζ*_C_*) of materials [17,18]:

(1) $$K_{C}=\frac{F_{T}}{\sqrt{2p\left(d\right)A(d)}}$$

(2) $$\zeta_{C}=\frac{K_{C}^{2}}{M}$$

where A(d) and $p\left(d\right)$ are the parameters related to the contact surface of the indenter with the meaterial, and the meanings of these parameters can be found in the literature [23]. M is the indentation modulus, which can be directly tested by the nanoindentation test.

As shown in Equation (1), the calculation of fracture toughness requires the $p\left(d\right)A(d)$ parameter, whose calculation method was introduced in the literature [17] and modified by Wei et al. [23,33]. Also, previous studies confirmed that the $p\left(d\right)A(d)$ is in proportion to the $d^{3}$ for the Berkovich tip [34], and the methodology for obtaining the proportionality coefficient between $p\left(d\right)A(d)$ and $d^{3}$ can be found in the literature [17,23,33]. In this study, the relationship between $p\left(d\right)A(d)$ and $d^{3}$ is shown in Equation (3), and it is assumed that this relationship remains constant for all nanoscratching tests in this study.

(3) $$p\left(d\right)A\left(d\right)=2.3014d^{3}$$

### 2.3. Nanoindentation Test

**Figure 2:**
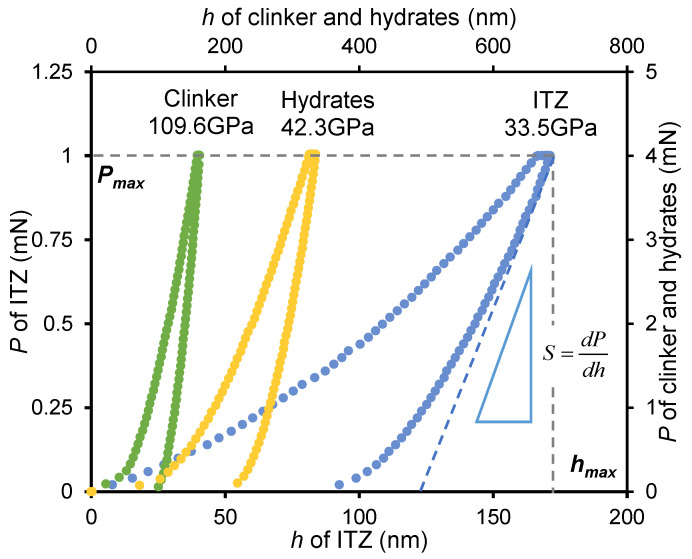
Typical load–indentation depth (*P–h*) curves for clinker, hydrates, and ITZ phases.

Nanoindentation testing was conducted by a KEYSIGHT G200 nano indenter in indentation mode with a Berkovich probe. The testing parameters for the nanoindentation test were shown in Table 3. Previous investigations [35,36] reported that the thickness of ITZ between clinker and hydration production in the unfrozen sample is typically below 2 μm. According to Durst et al. [37], reliable measurement of homogeneous mechanical properties for a specific phase requires maintaining an indentation depth-to-phase characteristic size ratio (h/D) below 0.1, which effectively minimizes interference from adjacent phases. Therefore, to accurately obtain the indentation modulus (*M*) of the ITZ phase, the maximum indentation depth was constrained to 200 nm. In this study, by using the testing parameter shown in Table 3, the maximum indentation depth was about 170 nm (as shown in Figure 2), thereby satisfying this essential measurement criterion for ITZ characterization.

Figure 2 shows the typical load–indentation depth curves obtained from testing the clinker, hydrates, and ITZ phases, and the indentation modulus (M) can be calculated by using Equation (4) [38]:

(4) $$M=\frac{\sqrt{\pi }}{2\beta }\frac{S}{\sqrt{A_{C}}}$$

where $S={\left(\frac{dP}{dh}\right)}_{h=h_{max}}$ denotes the contact stiffness, which is the slope of the initial unloading curve. *h_max_* denotes the maximum indentation depth. *β* denotes the geometrical correction factor, which can be set to 1.043 for the Berkovich probe. *P_max_* is the maximum load. *A_C_* denotes the projected area of contact, which depends on the contact depth [39]:

(5) $$A_{C}=24.5h_{c}^{2}$$

where *h_c_* denotes the contact depth, which is related to the maximum indentation depth and can be calculated as:

(6) $$\frac{h_{C}}{h_{max}}=1-0.75\frac{P_{max}}{S\cdot h_{max}}$$

### 2.4. SEM/BSE Test

In this study, the FEI QUANTA 200 (FEI Company, Hillsboro Oregon, OR, USA) focused ion beam scanning electron microscope (FIB-SEM) was used to identify the phase, to observe the micro morphology of the frost cement paste, to determine the starting location of nanoscratch test, and to observe the scratching path. The accelerating voltage was set to 15 kV, and the working distance was maintained at 10 mm.

**Figure 3:**
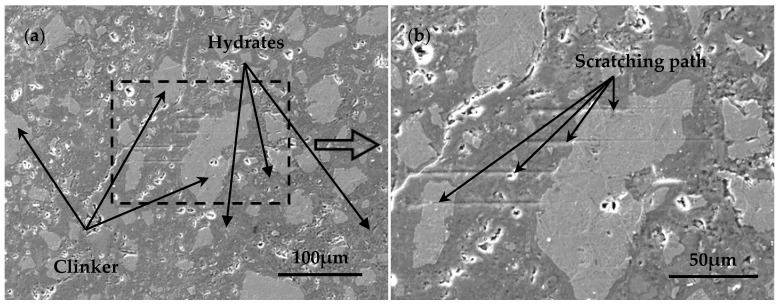
SEM images for (**a**) selecting scratching starting point and (**b**) observing scratching path.

The magnification of each SEM/BSE image should be determined based on the size of the target area being observed. When selecting the scratching location and analyzing the size of the clinker particles, it should be ensured that no large pores and cracks exist in the scratching path of the target clinker particles. The magnification was set to about 1500 times for selecting the location of the scratching, with an image size of about 390 μm × 289 μm and pixel size of 0.38 μm, as shown in Figure 3a. For observing the scratching path, because the length of each scratching was 100 μm, the magnification was set to be about 3000 times to ensure the entire scratching path was visible within the field of view and to maximize clarity. The image was about 185 μm × 137 μm, and the size of each pixel was 0.18 μm, as shown in Figure 3b.

### 2.5. Mercury Intrusion Porosimetry (MIP) Test

In this study, the Micromeritics AutoPore IV 9500 (Micromeritics, Norcross, GA, USA) was used to conduct the mercury intrusion porosimetry (MIP) test, aiming to study the effects of freezing age and nano-silica on the porosity of cementitious materials. Parameters such as porosity and pore distribution were utilized to assess the pore structure of the samples. It should be noted that the porosity measured in this study represents the total porosity of connected pores detectable by MIP and should not be confused with the total pore volume of the samples [33]. For the samples shown in Table 2, three cube pieces about 5 mm in size were prepared by the sawing method. Before the MIP tests, all the MIP samples were immersed in isopropanol solvent to stop hydration and then oven-dried at 60 °C until a constant mass was achieved. The MIP tests were conducted with a mercury contact angle of 130° and a maximum pressure of 420 MPa. All MIP tests were conducted at the age of 28 days.

## 3. Results and Analysis

### 3.1. Effect of Frost Attack on the Porosity of Cement Paste

**Figure 4:**
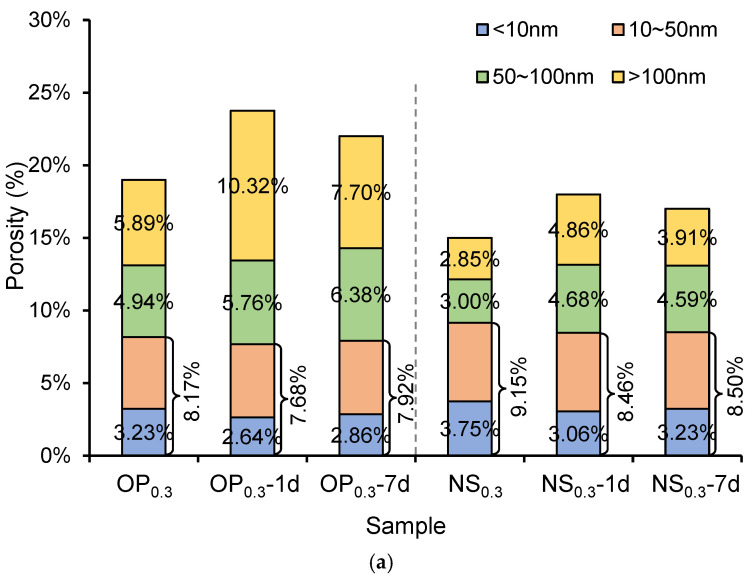

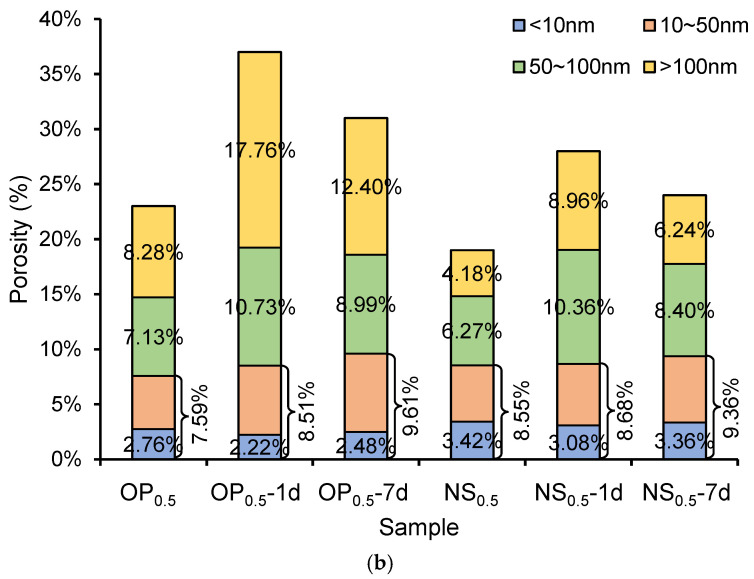
Porosity and pore size distribution of ordinary Portland cement pate sample (OP) and nano-silica-strengthened cement paste sample (NS) with water-to-cement (*w*/*c*) ratios of (**a**) 0.3 and (**b**) 0.5.

Figure 4a shows the porosity and pore size distribution of OP and NS samples with a *w*/*c* ratio of 0.3 at an age of 28 days under different freezing conditions. The results indicate that the early age freezing significantly increased the number of pores with a size larger than 100 nm and slightly reduced the number of pores with a size less than 50 nm. Early age freeze–thaw cycles inhibit the hydration of cement, and the expansion of free water under freezing conditions leads to the formation of larger pores, hindering the development of a denser microstructure. Moreover, some small pores may be enlarged due to the frost-induced expansion force, which increases the number of larger pores and decreases the number of small pores.

Compared to the 1d sample, the reduction in the number of large pores after freezing at 7 days is mainly attributed to the higher degree of hydration and the formation of a denser microstructure before freezing. At 7 days, the cement paste has developed a more continuous C–S–H gel network and contains less freezable water, which reduces the potential for frost-induced pore expansion. In addition, the partially hydrated clinker particles and existing hydration products limit ice formation and crack propagation. During the later curing period, residual hydration and self-healing effects at unfrozen interfaces further refine and fill the larger pores generated during early freezing.

In the NS sample, the number of pores with a size larger than 100 nm was significantly reduced, which was attributed to the incorporation of nano-silica, which facilitated the hydration reaction and filled the macropores, resulting in the formation of a denser structure cement paste. However, the nano-silica has limited effects on increasing the number of pores with a size less than 50 nm. This means that the hydrated calcium silicate generated by nano-silica only fills the pores with a size of larger than 100 nm rather than the pores with a size less than 50 nm. Meanwhile, since the minimum diameter of the nano-silica used in this study is 15 nm, the “filler effects” of nano-silica on the pores with a size less than 50 nm was not significant. Specifically, the pozzolanic reaction between nano-silica and calcium hydroxide (CH) generates additional C–S–H gel, which can fill and refine the medium-sized pores (50–100 nm) and improve the microstructure. However, due to the limited amount of nano-silica and slower pozzolanic reaction at low temperature, this effect is less pronounced for pores smaller than 50 nm.

Figure 4b shows the porosity and pore size distribution of OP and NS samples with a *w*/*c* ratio of 0.5 at the age of 28 days under different freezing conditions. Similarly, early age freezing significantly increased the number of pores with a size larger than 100 nm, and the incorporation of nano-silica reduced the number of pores with a size larger than 100 nm. Comparing Figure 4a and Figure 4b, it is revealed that the number of pores with a size less than 50 nm was not significantly increased in the paste with a larger *w*/*c* ratio. Therefore, the deterioration effect of freeze–thaw cycles on the microstructure of cementitious materials is mainly reflected in the increase in the number of pores with a pore size larger than 100 nm. Similarly, nano-silica could enhance the frost resistance and microstructure of cementitious materials by reducing the number of pores with a pore size larger than 100 nm.

### 3.2. Clinker Size Quantification by BSE Imaging

Previous studies showed that the size of the aggregate is positively correlated with the thickness of the surrounding ITZ. Correspondingly, the ITZ thickness between clinker and hydrates is also related to the size of clinker, therefore it is necessary to quantify the size of clinker.

**Figure 5:**
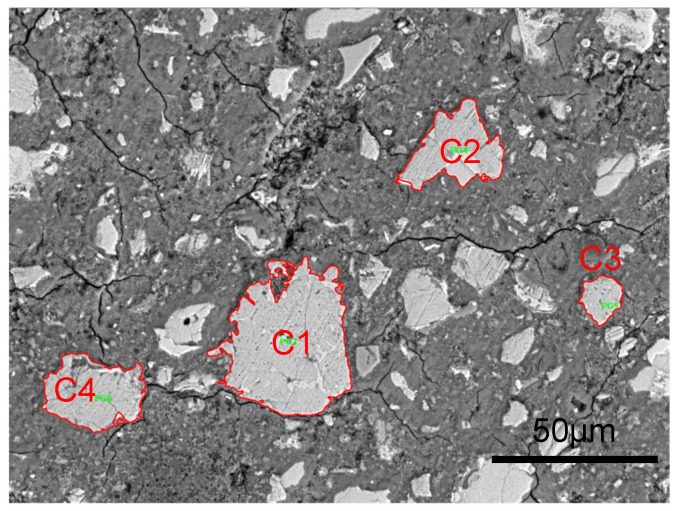
BSE images of OP_0.3_ sample at the age of 28 days under a magnification of 3000 times.

Taking the OP_0.3_ sample as an example to demonstrate the quantification method of the clinker size, based on the SEM/BSE testing method in Section 2.4, BSE images of the surface morphology of the OP_0.3_ sample were obtained under a magnification of 3000 times, as shown in Figure 5. The size of each pixel in the image is 0.18 μm × 0.18 μm. In Figure 5, clinker phases exhibit bright gray features and can be segmented preliminarily by using the Image Pro Plus software under the gray level of 150~255. For the boundary between the clinker phase and the hydrates phase, an “overflow” method was used to distinguish the boundary, the principle and steps of which are detailed in the literature [40]. After determining the boundaries of each clinker, the number of pixels occupied by each clinker can be counted by using the Image Pro Plus software, and then the equivalent diameter *D* of the clinker phase can be calculated by Equation (7). For the four clinkers shown in Figure 5, the gray level, pixel number, and equivalent diameter are summarized in Table 4.

(7) $$D=\sqrt{\frac{4n\cdot a^{2}}{\pi }}$$

where n is the pixel number of each clinker, and a is the size of each pixel.

### 3.3. Phase Identification by Nanoscratch Technique

#### 3.3.1. Fracture Toughness of Individual Phase

**Figure 6:**
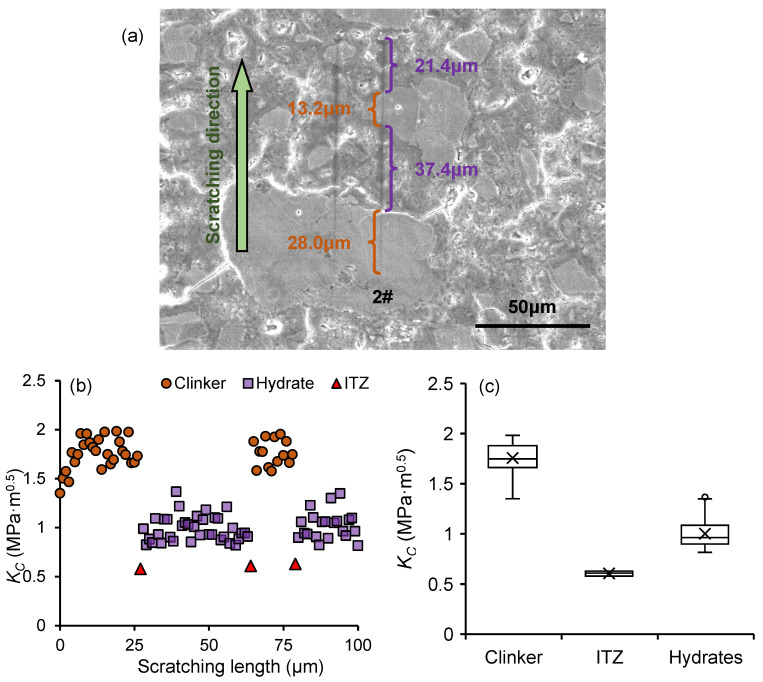
(**a**) SEM image of the scratching path, (**b**) the fracture toughness distribution along the scratching path, and (**c**) the fracture toughness of the individual phase.

Figure 6a shows the two scratching paths in the OP_0.3_ sample at the age of 28 days. Equations (1)–(3) were used to calculate the fracture toughness distribution along scratching path 2#, as shown in Figure 6b. For the phase thickness determination in Figure 6a, the pixel number of each phase was obtained by the distance measurement function in Image Pro Plus software, and the thickness of each phase was calculated by multiplying the pixel count by 0.18 μm (the pixel size). For the phase thickness determination in Figure 6b, the distance between two adjacent data points was 1 μm, and the phase thickness was quantified by the number of data points.

Comparing the SEM image shown in Figure 6a with the fracture toughness distribution shown in Figure 6b, the fracture toughness of each phase can be quantified. From the fracture toughness distribution shown in Figure 6b, the clinker phase, the hydrates phase, and the ITZ phase can be distinguished, with fracture toughness values of 1.57~2.01 MPa·m^0.5^, 0.81~1.12 MPa·m^0.5^, and 0.58~0.63 MPa·m^0.5^, respectively, as shown in Figure 6c. These results are consistent with those reported by Wei et al. [23] and Kong et al. [21].

#### 3.3.2. ITZ Thickness Quantification

Based on the fracture toughness of individual phase shown in Section 3.3.1, it is found that the fracture toughness of ITZ is the weakest, indicating that the ITZ is a critical factor influencing the fracture properties of frozen cement paste. Meanwhile, studies have demonstrated that the mechanical properties of ITZ are closely correlated with its thickness, which is of great significance for characterizing the development of ITZ thickness before and after freeze–thaw cycles.

By using the nanoscratch technology, Xu et al. [41] proposed an ITZ thickness quantification method based on the distribution of coefficient of friction (COF method). However, because the COF of the individual phase tested by nanoscratch technology may be affected by the surface roughness of the sample, the ITZ thickness quantified by COF method may lack accuracy. According to the study of Wei et al. [23], a *K_C_* method for calculating the ITZ thickness was proposed based on the fracture toughness distribution along the scratching path. As surface roughness has a lesser impact on the calculated mechanical properties compared to the COF, the ITZ thickness value calculated by the *K_C_* method is theoretically more accurate than that derived from the COF method.

The schematic of the ITZ thickness calculation process is shown in Figure 6. In Figure 6a, the fracture toughness at ITZ was taken as the symmetry center to calculate the symmetric *K_C_* (and symmetric average *K_C_*) of the hydrates phase and clinker phase. In Figure 6b, “S”-shaped fitting curves for the *K_C_* of the clinker and hydrate phases were constructed, incorporating both the tested *K_C_* and symmetric *K_C_* values. Then, a tangent line to the “S”-shaped fitting curve at the *K_C_* data point of the ITZ was plotted, ensuring it intersected with the two average *K_C_* lines (tested and symmetric lines). The ITZ thickness was determined by the distance between the intersection points, that is, *d* = (*d*_1_ + *d*_2_)/2, as shown in Figure 6b.

**Figure 7:**
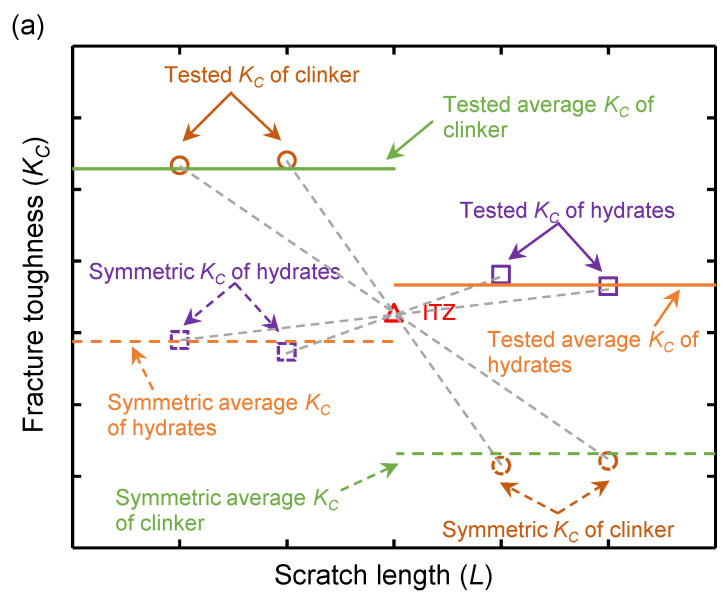

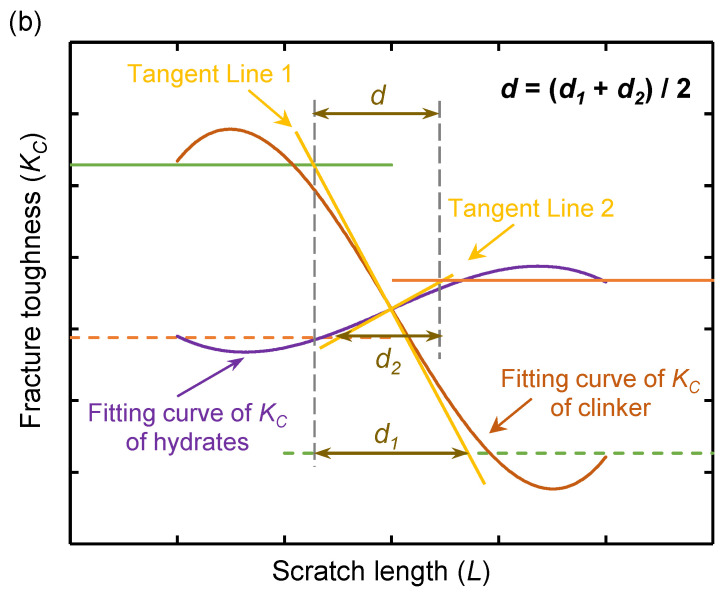
Process of *K_C_* method for calculating the thickness ITZ: (**a**) calculating the symmetric fracture toughness values of each phase; (**b**) drawing the tangent line of the fitting curve to intersect the average fracture toughness for determining the ITZ thickness.

**Figure 8:**
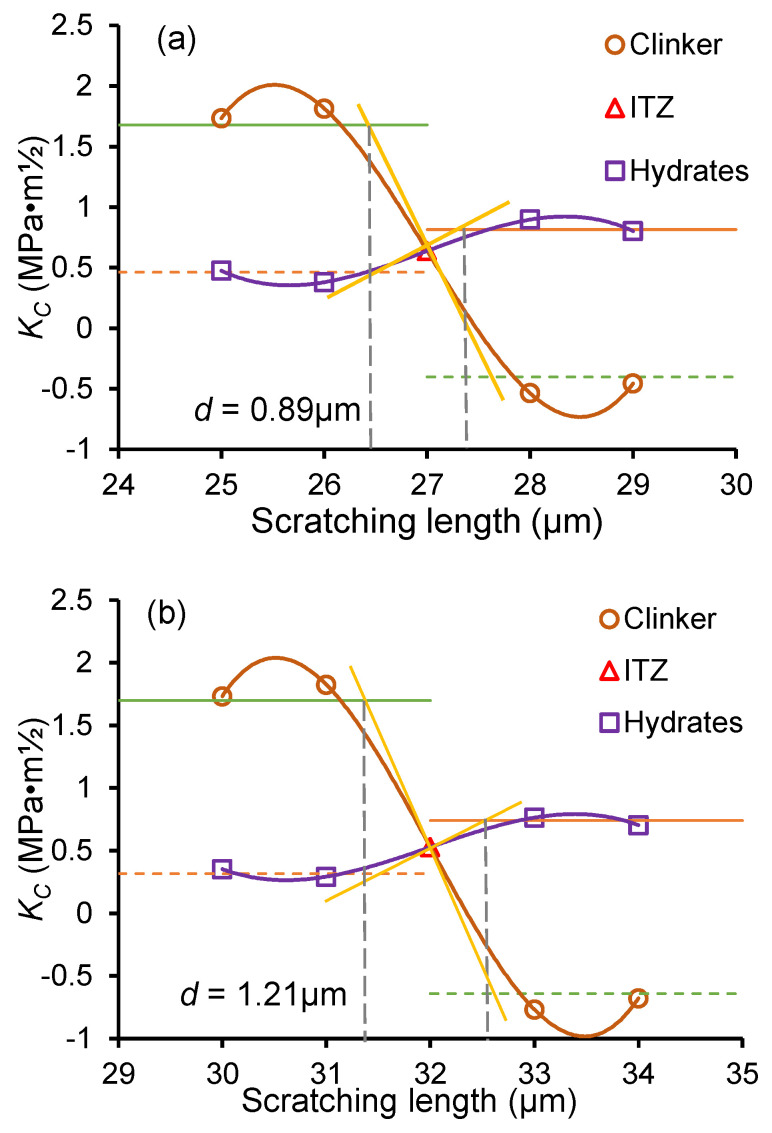

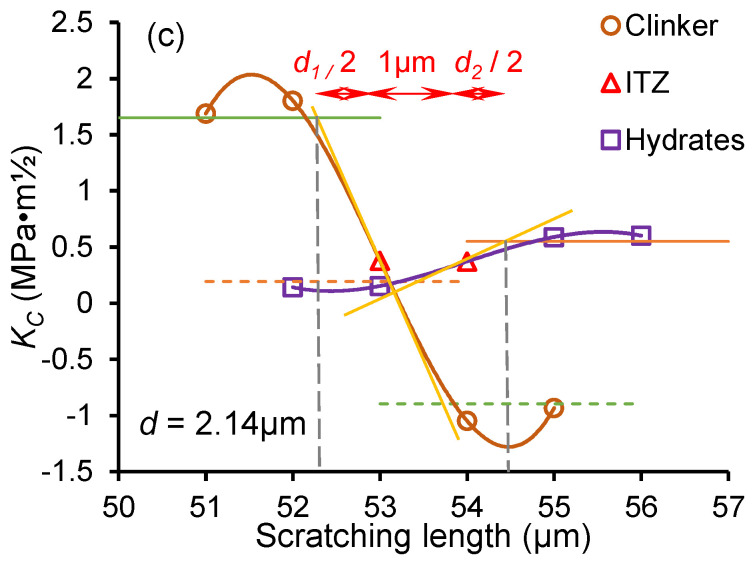
ITZ thickness calculation using *K_C_* method for (**a**) OP_0.3_ sample, (**b**) OP_0.3_-7d sample, and (**c**) 1 day (OP_0.3_-1d) with *w*/*c* ratio of 0.3.

Taking scratching 2# in Figure 6 as an example, the ITZ location was first identified using SEM images. The fracture toughness values of the nearby clinker and hydrate phases were extracted, and average values were calculated. Following the procedure in Figure 7, symmetry values were obtained and the tangent line drawn, allowing for calculation of the ITZ thickness. For the sample shown, the clinker equivalent diameter is 72.0 μm, and the ITZ thickness is 0.89 μm. Figure 8b,c show the calculation for the OP_0.3_-7d and OP_0.3_-1d samples. In cases with multiple ITZ fracture points, only two points near th eclinker and hydrate were used for curve fitting, with 1 μm spacing.

From Figure 8, the ITZ thicknesses of OP_0.3_-7d and OP_0.3_-1d are 1.21 μm and 2.14 μm, respectively. Early freeze–thaw cycles increase the ITZ thickness, with earlier frozen ages resulting in a thicker ITZ. This is due to the retardation of hydration at early ages. Freezing slows C–S–H generation and disrupts microstructure development in the ITZ. Later-age freezing allows for more mature hydration, producing a denser ITZ with higher fracture toughness, thus limiting thickness growth.

### 3.4. Effect of Frost Attack on the Fracture Properties and Thickness of ITZ

As the weakest solid phase in cement-based materials, the ITZ serves as the primary source of crack initiation and propagation and plays a critical role in affecting the fracture performance of cement-based materials. During the early hydration process of cement, ITZ gradually transitions from a porous structure to a denser one, increasing its own strength and thus improving the fracture performance of cement-based materials. Therefore, studying the effect of early freeze–thaw cycles on the fracture properties of ITZ is of great significance for understanding the mechanisms of frost damage and crack propagation.

**Figure 9:**
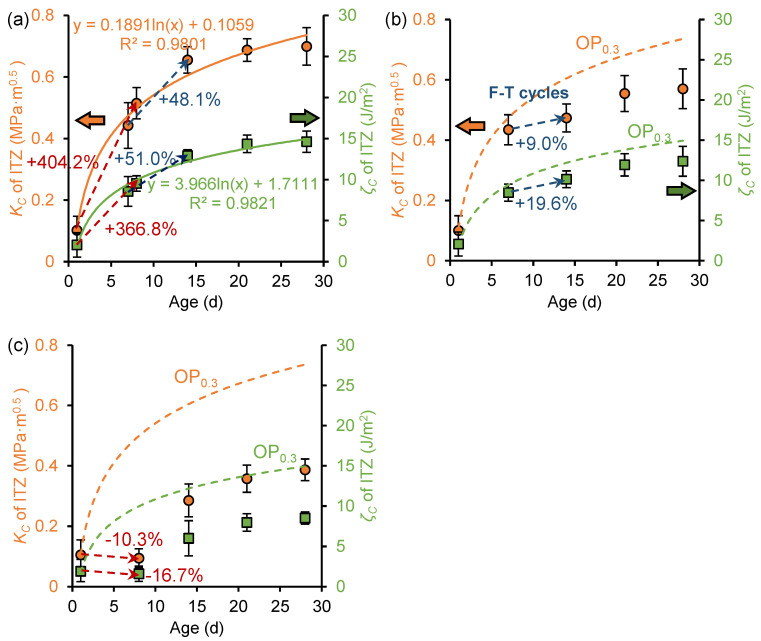
Development of fracture toughness (*K_C_*) and fracture energy (*ζ_C_*) of ITZ in (**a**) OP_0.3_ sample, (**b**) OP_0.3_-7d sample, and (**c**) OP_0.3_-1d sample.

Figure 9 shows the evolution of fracture toughness and fracture energy of ITZ before and after freeze–thaw cycles in the OP_0.3_, OP_0.3_-7d, and OP_0.3_-1d samples. For the unfrozen OP_0.3_ sample, the fracture toughness and fracture energy at age 28 days were 0.70 MPa·m^0.5^ and 14.61 J/m^2^, respectively, consistent with the findings reported by Kong et al. [21]. In addition, the fracture toughness and fracture energy exhibit a logarithmic relationship with curing age. This has the same development trend as the macroscopic fracture performance [21], indicating that the macrofracture properties of cement paste can be demonstrated at the microscale.

Comparing Figure 9a with Figure 9b,c, it is revealed that early freeze–thaw cycles inhibit the development of ITZ fracture toughness and fracture energy. At the age of 28 days, the fracture toughness and fracture energy of OP_0.3_-7d samples were 0.57 MPa·m^0.5^ and 12.36 J/m^2^, respectively, which decreased by 18.4% and 15.4% compared to OP0.3 samples. The fracture toughness and fracture energy of the OP_0.3_-1d sample were 0.39 MPa·m^0.5^ and 8.51 J/m^2^, respectively, which decreased by 44.7% and 41.8% compared to the OP_0.3_ sample. This indicates that earlier freezing ages result in more pronounced inhibition of fracture performance development. Specifically, for the OP_0.3_-1d sample, the fracture toughness and fracture energy showed negative growth during freezing, attributed to incomplete hydration reactions and insufficient paste strength at the age of 1 day. Freezing and thawing cause the expansion of this part of the water, suppressing hydration reactions and damaging the microstructure of the paste. After the freeze–thaw cycles were completed, the fracture toughness and fracture energy of the sample continued to develop during the secondary curing period, but could not fully recover to the levels observed in unfrozen samples. The fracture properties damage caused by freeze–thaw cycles was partially irreversible, and timely and appropriate curing after the end of freeze–thaw can repair the frost damage to a certain extent.

**Figure 10:**
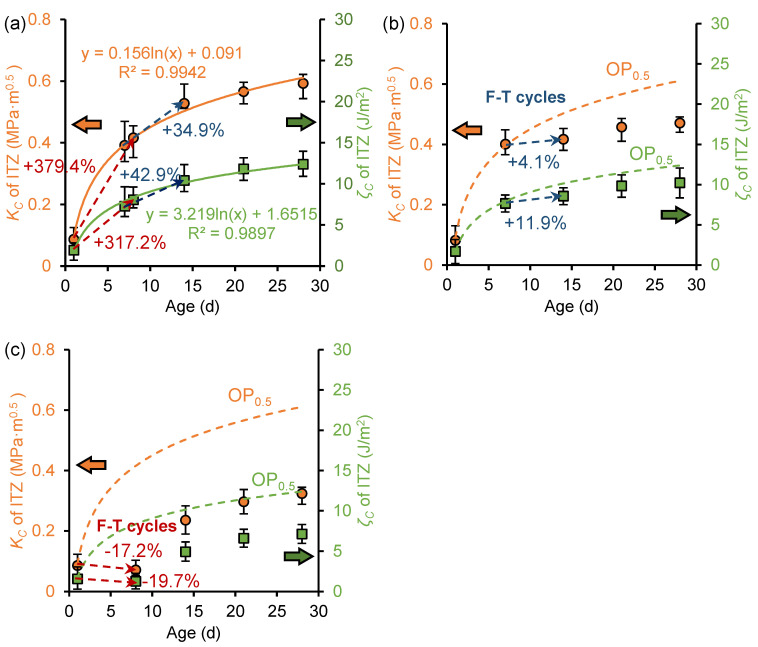
Development of fracture toughness (*K_C_*) and fracture energy (*ζ_C_*) of ITZ in (**a**) OP_0.5_ sample, (**b**) OP_0.5_-7d sample, and (**c**) OP_0.5_-1d sample.

Figure 10 illustrates the development of fracture toughness and fracture energy of the ITZ phase before and after freeze–thaw cycles in the OP_0.5_, OP_0.5_-7d, and OP_0.5_-1d samples. For the OP_0.5_ sample, the fracture toughness and fracture energy also exhibit a logarithmic relationship with curing age. At the age of 28 days, the fracture toughness and fracture energy of the OP_0.5_ sample were 0.59 MPa·m^0.5^ and 12.4 J/m^2^, respectively, lower than those of the OP_0.3_ sample. An increase in the *w*/*c* ratio reduced the fracture properties of cement paste. Comparing Figure 9 to Figure 10, it can be found that the paste with a *w*/*c* ratio of 0.5 experienced greater damage to its fracture toughness and fracture energy during freezing. This is attributed to the higher water content in pastes with larger *w*/*c* ratios, which generates greater freezing expansion forces during freezing, preventing the formation of a dense structure and leading to more severe deterioration of the fracture properties of the ITZ. During the secondary curing period, the fracture properties of the paste with a *w*/*c* ratio of 0.5 also continued to develop. At the age of 28 days, the fracture toughness and fracture energy of OP_0.5_-7d decreased by 21.5% and 18.5%, respectively, compared to the OP_0.5_ sample, and the fracture toughness and fracture energy of OP_0.5_-1d decreased by 46.3% and 43.4%, respectively, compared to the OP_0.5_ sample. Compared to the reduction in fracture properties of frozen paste with a *w*/*c* ratio of 0.3 at 28 days of age, early age freeze–thaw cycles were found to have more pronounced effects on the fracture properties of high *w*/*c* ratio cement paste.

**Figure 11:**
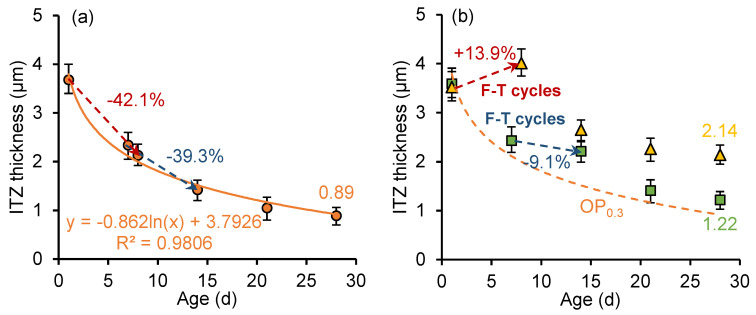
ITZ thickness development of (**a**) OP_0.3_ sample and (**b**) OP_0.3_-7d sample and OP_0.3_-1d sample.

**Figure 12:**
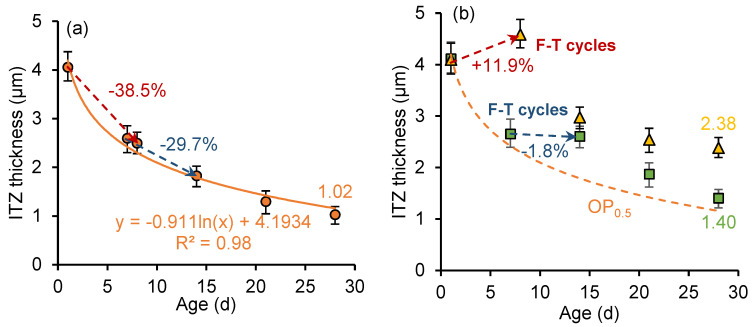
ITZ thickness development of (**a**) OP_0.5_ sample and (**b**) OP_0.5_-7d sample and OP_0.5_-1d sample.

Figure 11 and Figure 12 show the development of the ITZ thickness in the cement paste with a *w*/*c* ratio of 0.3 and 0.5, respectively. For the unfrozen paste, the thickness of ITZ develops logarithmically with curing age. The ITZ thickness in the sample with a *w*/*c* ratio of 0.3 is smaller than that in the sample with a *w*/*c* ratio of 0.5, and the rate of ITZ thickness development is higher. However, early age freeze–thaw cycles slow the formation of denser (narrower) structures in the ITZ. Particularly in samples frozen at the age of 1 day, an increase in ITZ thickness is observed during freezing. In addition, secondary curing positively influences the development of ITZ thickness, with the ITZ thickness continuing to decrease during the secondary curing process.

In the early stage of hydration, the silicon components in the cementitious material can form loosely structured and porous hydration products through hydration reactions, which rapidly deposit on the surface of clinker particles. Meanwhile, the calcium hydroxide (CH) crystals tend to form a layered structure and adhere to the surface of clinker particles. As the hydration process continues, the generated calcium silicate hydrate (C-S-H) and smaller CH crystals gradually fill the pores, leading to increased ITZ density, reduced thickness, and improved mechanical properties. However, early age freeze–thaw cycles slowed down the hydration reaction rate and disrupted the development of the microstructure of ITZ. Meanwhile, freeze–thaw cycles increase the porosity of cement paste and hinder the growth of ITZ compactness.

### 3.5. Effect of Nano-Silica on the Fracture Properties and Thickness of ITZ

**Figure 13:**
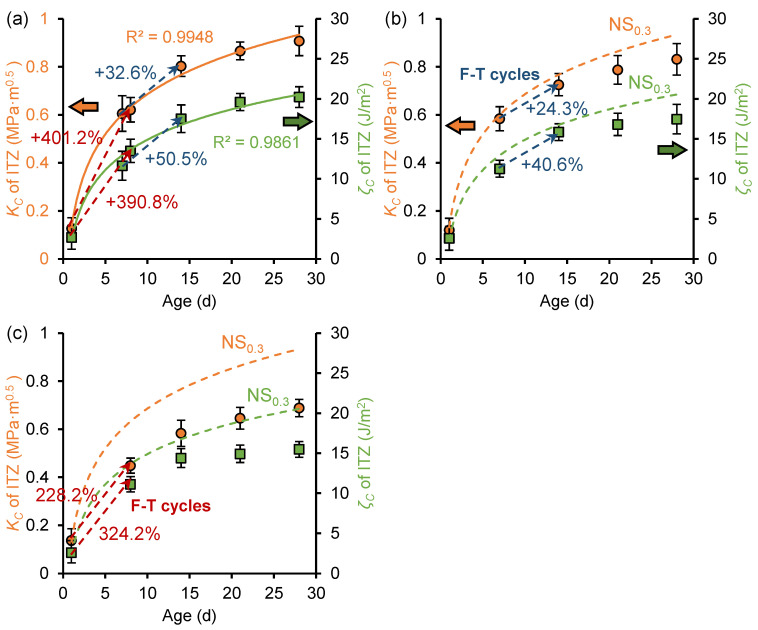
Development of fracture toughness (*K_C_*) and fracture energy (*ζ_C_*) of ITZ in (**a**) NS_0.3_ sample, (**b**) NS_0.3_-7d sample, and (**c**) NS_0.3_-1d sample.

Figure 13 shows the development of fracture toughness and fracture energy of the ITZ before and after freeze–thaw cycles in NS_0.3_, NS_0.3_-7d, and NS_0.3_-1d samples. For NS_0.3_ samples that have not been frozen, the fracture performance also exhibits a logarithmic increase with curing age. Compared to the OP sample, at the age of 28 days, the fracture toughness of the ITZ in NS_0.3_, NS_0.3_-7d, and NS_0.3_-1d samples increased by 29.8% (0.91 MPa·m^0.5^), 45.7% (0.83 MPa·m^0.5^), and 78.0% (0.69 MPa·m^0.5^), respectively, and the fracture energy increased by 38.4% (20.22 J/m^2^), 41.3% (17.47 J/m^2^), and 82.1% (15.49 J/m^2^), respectively. The nano-silica significantly improves the fracture performance of cement paste, particularly in early age frozen paste.

The development of ITZ fracture properties were also improved by the nano-silica during freeze–thaw cycles. For example, the fracture toughness and fracture energy of NS_0.3_-1d samples increased by more than twofold and threefold, respectively, during freeze–thaw cycles, but the fracture properties of OP_0.3_-1d samples exhibited negative growth during freeze–thaw cycles. This is attributed to the ability of nano-silica to accelerate the early hydration rate, leading to the generation of additional C-S-H through pozzolanic reactions compared to ordinary cement paste [42]. C-S-H is the primary material responsible for the mechanical properties of cement paste, and its increased presence in NS samples contributes to their higher fracture toughness. Meanwhile, the accelerated early hydration reactions consume more free water, further reducing the frost damage.

**Figure 14:**
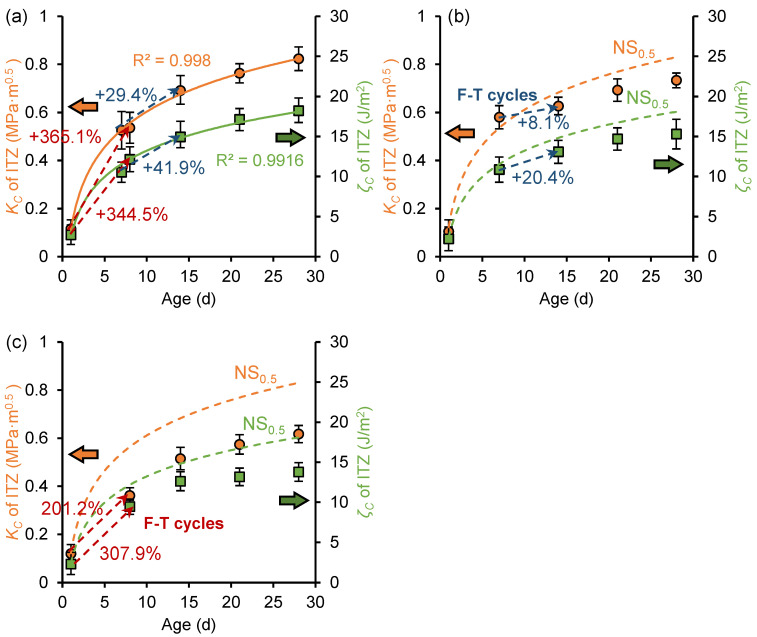
Development of fracture toughness (*K_C_*) and fracture energy (*ζ_C_*) of ITZ in (**a**) NS_0.5_ sample, (**b**) NS_0.5_-7d sample, and (**c**) NS_0.5_-1d sample.

Figure 14 shows the development of fracture toughness and fracture energy of ITZ before and after freeze–thaw in the NS_0.5_, NS_0.5_-7d, and NS_0.5_-1d samples. Compared to the fracture properties development characteristics of the OP_0.5_ sample shown in Figure 10, during the freeze–thaw period, the NS_0.5_ sample exhibits a significantly higher growth rate in fracture properties, with the 1-day-old frozen paste showing greater improvement than the 7-day-old frozen paste. This indicates that nano-silica also significantly improves the frost resistance of high *w*/*c* ratio cement paste, with earlier freezing ages resulting in more pronounced improvements. Compared to the fracture properties development characteristics of the NS_0.3_ sample shown in Figure 13, the 0.3 *w*/*c* ratio cement paste exhibits a slightly higher growth rate in fracture performance during the same freezing period than the 0.5 *w*/*c* ratio cement paste, indicating that nano-silica has a more significant effect on improving the frost resistance of low *w*/*c* ratio paste.

**Figure 15:**
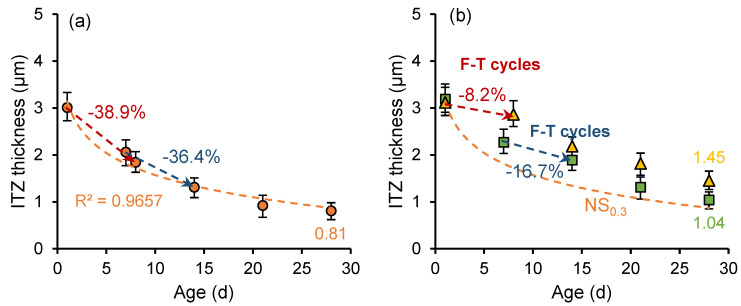
ITZ thickness development of (**a**) NS_0.3_ sample and (**b**) NS_0.3_-7d sample and NS_0.3_-1d sample.

**Figure 16:**
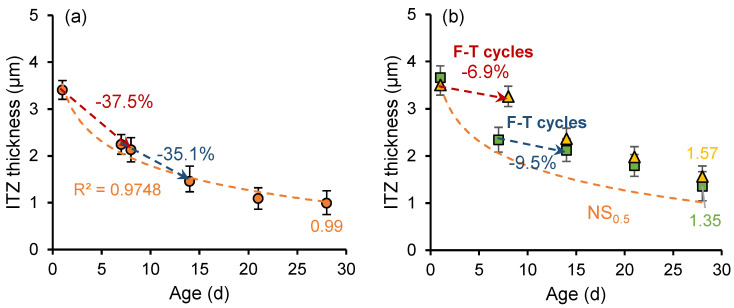
ITZ thickness development of (**a**) NS_0.5_ sample and (**b**) NS_0.5_-7d sample and NS_0.5_-1d sample.

Figure 15 and Figure 16 illustrate the development of ITZ thickness in nano-silica-enhanced cement paste (NS sample) with *w*/*c* ratios of 0.3 and 0.5. Compared to the OP sample with a *w*/*c* ratio of 0.3, at the age of 28 days, the ITZ thickness of unfrozen paste (OP_0.3_), the 7-day-age frozen paste (OP_0.3_-7d), and the 1-day-age frozen paste (OP_0.3_-1d) decreased by 8.99%, 14.75%, and 32.03%, respectively. The ITZ thickness of unfrozen paste (OP_0.5_), 7-day-old frozen paste (OP_0.5_-7d), and 1-day-old frozen paste (OP_0.5_-1d) with a water cement ratio of 0.5 decreased by 3.38%, 17.63%, and 34.25%, respectively. Nano-silica has a more pronounced effect on reducing the ITZ thickness of early frozen paste with high *w*/*c* ratio. The addition of nano-silica to cement paste results in the formation of a denser microstructure. Moreover, the particle diameter of nano-silica is about three orders of magnitude smaller than that of cement particles (nm vs. μm), enabling it to act as a filler material that enhances the density of cement paste [43]. The combination of two effects results in a lower thickness of the ITZ in cement paste incorporating nano-silica.

**Figure 17:**
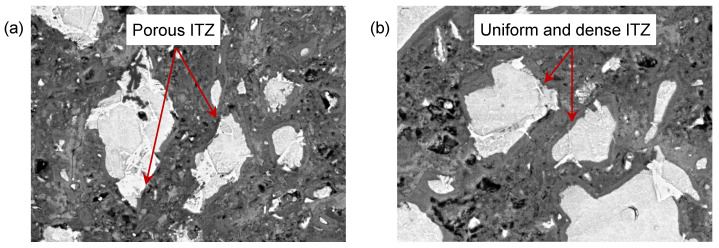
BSE image of (**a**) OP_0.3-7d_ paste sample and (**b**) NS_0.3-7d_ paste sample at the age of 28 days.

Figure 17 shows the BSE images of the OP_0.3-7d_ and NS_0.3-7d_ samples at the age of 28 days. Compared to the porous ITZ phase in the OP_0.3-7d_ sample, the ITZ phase in NS_0.3-7d_ has a more homogeneous and denser microstructure. This phenomenon reflects the improvement of nano-silica on the microstructure of the ITZ phase. Furthermore, the ITZ phase with a denser microstructure has a narrower thickness and higher mechanical properties.

## 4. Discussion

### 4.1. Relationship Between ITZ Thickness and Fracture Properties

ITZ is the weakest phase in cement-based materials, and the thickness of ITZ directly affects the fracture toughness and crack resistance of the material. A thicker ITZ is usually accompanied by a higher porosity and lower density, making it a primary site for crack initiation and propagation, which consequently diminishes the overall fracture properties of the material. Studying the relationship between ITZ thickness and fracture properties can provide a theoretical basis for improving the fracture toughness of materials.

**Figure 18:**
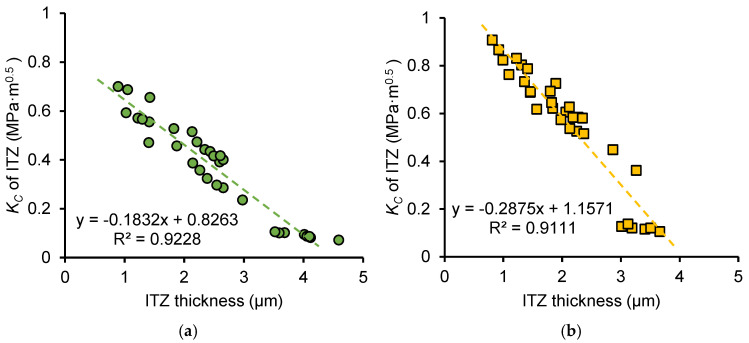
Relationship between fracture toughness of ITZ and thickness of ITZ for (**a**) OP sample and (**b**) NS sample.

**Figure 19:**
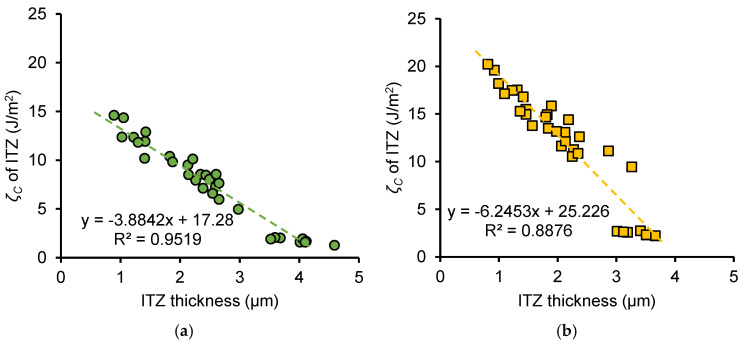
Relationship between fracture energy of ITZ and thickness of ITZ for (**a**) OP sample and (**b**) NS sample.

Figure 18 shows the relationship between ITZ fracture toughness and thickness, and Figure 19 shows the relationship between ITZ fracture energy and thickness for OP and NS samples. Data include *w*/*c* ratios of 0.3 and 0.5 and different frozen ages. For both OP and NS samples, fracture properties decrease linearly with increasing ITZ thickness. This is because thicker ITZs contain a less dense network of hydration products and weaker C–S–H formation, reducing local fracture toughness and energy.

For the same ITZ thickness, NS samples exhibit higher fracture properties than OP samples. This improvement arises from the combined filler effect and pozzolanic reaction of nano-silica. Nano-silica fills part of the capillary pores and reacts with CH to produce additional C–S–H gel, densifying the ITZ even at larger thicknesses. This enhances both the fracture toughness and fracture energy compared with ordinary paste.

Moreover, according to the fitting equations shown in Figure 18, assuming an ITZ thickness of zero, the fracture toughness of the OP sample is 0.83 MPa·m^0.5^ and the fracture toughness of the NS sample is 1.16 MPa·m^0.5^, which is similar to the fracture toughness of the hydrates phase in the OP and NS samples (0.89 MPa·m^0.5^ for hydrates phase of OP sample reported by Kong et al. [21] and 1.10 MPa·m^0.5^ for hydrates phase of NS sample reported by Wei et al. [30]). This implies that the ITZ is homologous to the hydrates, but when C-S-H gels accumulate around clinker particles, the C-S-H gels fair to form a dense phase comparable to the hydrates phase, leading to the formation of ITZ. Therefore, enhancing the fracture performance of the ITZ requires strategies to promote a denser microstructure within the paste and minimize ITZ thickness. Similarly, for the fitting equations of fracture energy with ITZ thickness shown in Figure 19, assuming an ITZ thickness of zero, the values of 17.28 J/m^2^ and 25.23 J/m^2^ also correspond to the fracture energy of hydrates in OP and NS samples.

### 4.2. Relationship Between Clinker Size and ITZ Properties

The ITZ is the weakest phase in the cementitious materials, and the thickness and mechanical characteristic of ITZ can be significantly affected by the clinker particles variation, which further affects the macromechanical properties of cementitious materials. Understanding the relationship between the size of clinker particles and the ITZ properties can help optimize the process and degree of hydration, improve the crack resistance of cement-based materials, and guide the development of high-performance materials.

**Figure 20:**
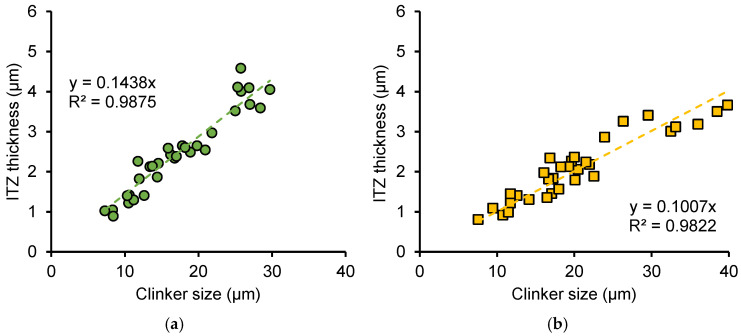
Relationship between thickness of ITZ and clinker size for (**a**) OP sample and (**b**) NS sample.

Figure 20a,b shows the relationship between the equivalent diameter of clinker particles and the thickness of the ITZ in OP and NS samples, respectively. Each figure includes data for samples with *w*/*c* ratios of 0.3 and 0.5 as well as frozen samples at different ages. It can be observed that the size of clinker particles is positively correlated with ITZ thickness, and the ITZ thickness increases linearly with the clinker particle size. For both ordinary cement paste (OP) and nano-silica-reinforced cement paste (NS), the proportional relationship between the size of clinker particles and the ITZ thickness was not affected by the early freeze–thaw cycles. This indicates that the increase in ITZ thickness caused by freeze–thaw cycles results from incomplete hydration reactions of cement particles, leading to larger residual cement particle sizes and, consequently, greater ITZ thickness. This indicates that the increase in ITZ thickness caused by larger clinker particles is closely related to the hydration reaction rate and diffusion mechanisms. Larger clinker particles have a lower specific surface area, leading to slower hydration at the particle surface. Moreover, ions must diffuse over longer distances from the clinker surface to the surrounding paste, delaying the formation of C–S–H in the ITZ and resulting in a thicker, less dense ITZ.

Comparing Figure 20a and Figure 20b, it is found that that for the same clinker particle size, the ITZ thickness in the NS sample is lower than that in the OP sample. This further demonstrates that the addition of nano-silica promotes the formation of a denser microstructure in the paste, thereby reducing the ITZ thickness.

**Figure 21:**
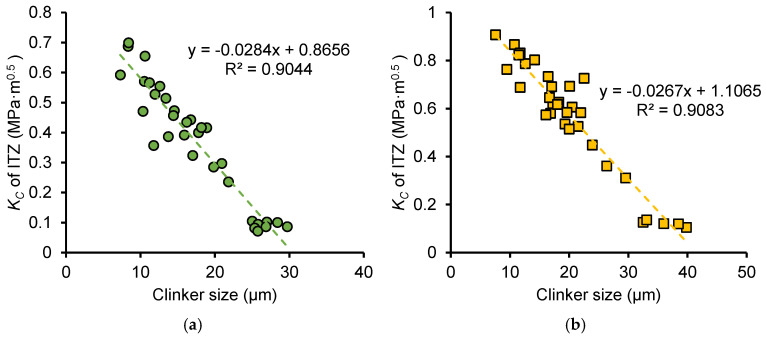
Relationship between fracture toughness of ITZ and clinker size for (**a**) OP sample and (**b**) NS sample.

Figure 21a,b shows the relationship between the equivalent diameter of clinker particles and the fracture toughness of the ITZ in OP and NS samples, respectively. Similarly, each figure includes data for samples with *w*/*c* ratios of 0.3 and 0.5 as well as frozen samples at different ages. It can be observed that a negative linear relationship exists between clinker particle size and ITZ fracture toughness. Larger clinker sizes correspond to ITZ phases with lower fracture toughness, and the fracture toughness of ITZ in NS samples is higher than that in OP samples when the ITZ thickness is the same. This further highlights the enhancing effect of nano-silica on the fracture toughness of ITZ.

Furthermore, based on extensive nanoscratch tests on clinker particles of varying sizes, the equations describing the relationships between ITZ thickness and fracture properties, as well as between clinker size and ITZ fracture toughness (provided in Figure 20 and Figure 21), can be used to predict ITZ thickness and fracture performance without requiring complex testing, provided clinker particle size data is obtained through simple SEM techniques.

### 4.3. Comparison Between Micro and Macrofracture Properties

**Figure 22:**
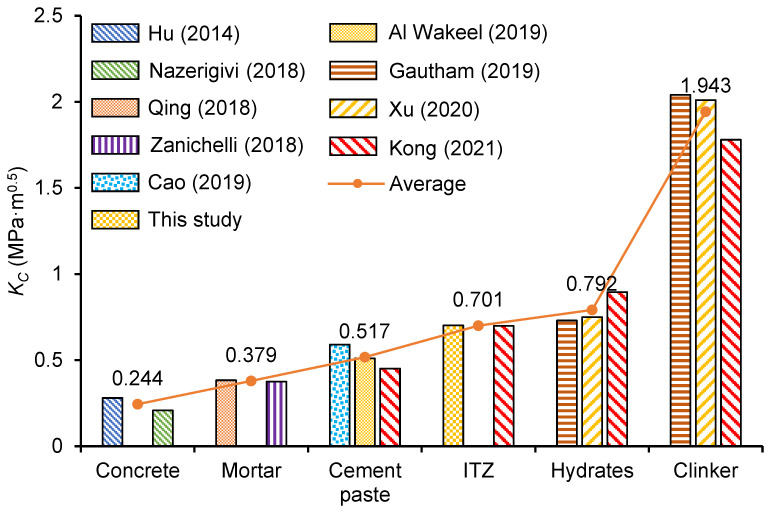
Fracture toughness of concrete at multiscale [21,46,47,48,49,50,51,52,53].

Cementitious materials, as multiscale materials, are non-homogeneous at different scales [44]. It is widely accepted in the literature that cementitious materials can be categorized into four distinct scales [45]. Figure 22 summarizes the fracture toughness of concrete, mortar, cement paste, and the micro phase tested in this study and from the literature [21,46,47,48,49,50,51,52,53]. The macrofracture toughness of concrete, mortar, and cement paste in the literature was measured by the three-point bending test, and the microfracture toughness of clinker particles and hydrates in the literature was measured by the nanoindentation and nanoscratch tests. It can be found that the fracture toughness of the materials progressively increases as the scale decreases, with fracture toughness values of 0.224 MPa·m^0.5^ for concrete, 0.379 MPa·m^0.5^ for mortar, 0.517 MPa·m^0.5^ for cement paste, and 0.792 MPa·m^0.5^ for hydrates. This is because, with the exception of solid phases such as the hydrates phase within the matrix, micropores and microcracks are also extensively distributed across all scales. These microcracks and pores facilitate the crack propagation and make the fracture toughness of cement paste materials decrease scale by scale.

## 5. Conclusions

Concrete structures in cold regions are exposed to prolonged freeze–thaw cycles, leading to microstructural deterioration and reduced mechanical properties and durability. Freezing and thawing induce pore water freezing and expansion, which contributes to the expansion of interfacial transition zone (ITZ) cracks and reduction in the fracture toughness of the material. As the weakest solid phase, ITZ is crucial for crack initiation and propagation, and the fracture properties of ITZ directly affect the overall durability of the material. Therefore, investigating the effects of freeze–thaw cycles on the microstructure and fracture properties of ITZ and exploring frost resistance enhancement methods are crucial for optimizing the durability design of concrete. In this study, the nanoscratch technique, MIP test, and SEM analysis were used to reveal the frost deterioration mechanism of ITZ from the perspective of microfracture mechanics and to explore the effects of nano-silica on the enhancement of frost resistance properties, aiming to provide theoretical support for the development of high-durability cementitious materials. The main conclusions are as follows:

(1) As the weakest phase in cementitious materials, the ITZ is significantly and negatively affected by freeze–thaw cycles in terms of fracture property and microstructural development, and the negative effects are more pronounced for pastes subjected to earlier freezing and with high *w*/*c* ratios. For the cement pastes frozen at the age of 1 day, the fracture properties showed a negative growth during the freezing period. Although secondary curing after freezing and thawing will alleviate the frost damage, it is also recommended to impose strict insulation in engineering.
(2) The thickness of ITZ can be significantly reduced and the fracture properties of ITZ can be enhanced by nano-silica. This is attributed to the ability of nano-silica to reduce the number of pores larger than 100 nm through enhanced hydration reactions and its filler effect, which makes the cement paste form a denser structure. The enhancement effect of nano-silica on the frost resistance of cementitious materials is more pronounced in materials with low *w*/*c* ratios.
(3) This is the first time to characterize the relationship between clinker particle size and thickness and fracture properties of ITZ using a combination of nanoscratch and SEM techniques. A significant negative correlation was observed between ITZ thickness and fracture properties, with thicker ITZs exhibiting lower fracture toughness and fracture energy. Moreover, clinker size also showed a linear relationship with both ITZ thickness and fracture properties. Larger clinker particles are associated with greater ITZ thickness and reduced fracture properties. For a given cement paste, neither the *w*/*c* ratio nor early age freeze–thaw cycles alter the linear relationship between clinker size and ITZ properties.

## Figures and Tables

**Table 1:** Properties of cement and NS particles.

Materials	Mass Fraction/%									Specific Gravity	Specific Surface Area/(m^2^ kg^−1^)
	SiO_2_	Al_2_O_3_	Fe_2_O_3_	CaO	MgO	SO_3_	Na_2_O	K_2_O	LOI		
Cement	20.55	4.59	3.27	62.59	2.61	2.93	0.53	0.83	1.77	3.14	350
Nanomaterials	Diameter			Specific surface area			Purity			Bulk density	PH value
Nano-silica	15 nm			250 m^2^/g			99.9%			0.1–0.15 g/cm^3^	5–7

**Table 2:** Proportioning and naming of the samples.

Sample	Cement	Water	Nano-Silica	Content by Weight	*w*/*c*	Frozen Age
OP_0.3_	1400	420	0	0	0.3	—
OP_0.3_-1d						1 day
OP_0.3_-7d						7 days
OP_0.5_	1400	700	0	0	0.5	—
OP_0.5_-1d						1 day
OP_0.5_-7d						7 days
NS_0.3_	1372	420	28	2%	0.3	—
NS_0.3_-1d						1 day
NS_0.3_-7d						7 days
NS_0.5_	1372	700	28	2%	0.5	—
NS_0.5_-1d						1 day
NS_0.5_-7d						7 days

**Table 3:** Testing parameters used for nanoindentation test.

Tested Phase	Maximum Load (mN)	Loading Rate (mN/s)	Holding Time (s)	Unloading Rate (mN/s)
Clinker	4	0.4	5	0.4
Hydrates	4	0.4	5	0.4
ITZ	1	0.1	5	0.1

**Table 4:** Gray level, pixel number, and equivalent diameter of each clinker in Figure 4.

Clinker Number	Gray Level Range	Pixel Number	Equivalent Diameter D (μm)
C1	156~255	34,530	37.7
C2	148~255	12,595	22.8
C3	161~255	3515	12.1
C4	152~255	12,786	23.0

## Data Availability

The original contributions presented in this study are included in the article. Further inquiries can be directed to the corresponding author.
